# 9.1% efficient zinc oxide/silicon solar cells on a 50 μm thick Si absorber

**DOI:** 10.3762/bjnano.12.60

**Published:** 2021-07-21

**Authors:** Rafal Pietruszka, Bartlomiej S Witkowski, Monika Ozga, Katarzyna Gwozdz, Ewa Placzek-Popko, Marek Godlewski

**Affiliations:** 1 Institute of Physics, Polish Academy of Sciences, Aleja Lotnikow 32/46, PL-02668 Warsaw, Poland; 2 Department of Quantum Technologies, Faculty of Fundamental Problems of Technology, Wroclaw University of Science and Technology, Wybrzeze Wyspianskiego 27, 50-370 Wroclaw, Poland

**Keywords:** atomic layer deposition, hydrothermal method, photovoltaics, silicon, solar cell, zinc oxide

## Abstract

Today, silicon solar cells (amorphous films and wafer-based) are a main source of green energy. These cells and their components are produced by employing various technologies. Unfortunately, during the production process, chemicals that are harmful for the environment and for human life are used. For example, hydrofluoric acid is used to texture the top electrode to improve light harvesting. In this work, and also in recent ones, we report a way to obtain 3D textures on the top electrode by using zinc oxide nanorods. The efficiency of a textured solar cell structure is compared with the one obtained for a planar zinc oxide/silicon structure. The present results show the possibility to produce efficient solar cells on a relatively thin 50 μm thick silicon substrate. Solar cells with structured top electrodes were examined by numerous measuring techniques. Scanning electron microscopy revealed a grain-like morphology of the magnesium-doped zinc oxide film. The size of the grains is closely related to the structure of the nanorods. The external quantum efficiency of the cells was measured. The obtained solar cell shows response in a wide spectral range from ultraviolet to infrared. Current–voltage and current–voltage–temperature measurements were performed to evaluate basic photovoltaic parameters. At room temperature, the cells efficiency equals to 9.1% for textured structures and 5.4% for planar structures, respectively. The work, therefore, describes an environmentally friendly technology for PV architecture with surface textures increasing the efficiency of PV cells.

## Introduction

The constantly increasing emission of greenhouse gases creates a growing environmental problem. Burning of fossil fuels leads to an increase of the amount of carbon dioxide in the atmosphere. The effect of this activity is global warming [1]. Fortunately, public awareness of the ecological problem opens the way to the installation of “green” (i.e., environmentally friendly) sources of energy. Moreover, such sources are now economically justified, which is due to impressive reduction of their costs. Currently, the most important sources of green energy are photovoltaics (PV), wind generators, and hydropower installations. Of these energy sources, the photovoltaics market is developing extremely dynamically. The growing interest in photovoltaics results not only in large installations (large solar farms), but also in small solarpower systems installed on residential buildings.

According to a report prepared by the Fraunhofer Institute (Germany), the solar cells (SCs) market is dominated by silicon [2]. In 2019, monocrystalline, polycrystalline, and amorphous silicon accounted for about 95% of the PV market. The highest (laboratory device) efficiency for monocrystalline and polycrystalline silicon cells equals to 26.7% and 22.3%, respectively. For solar cell modules available on the market, the efficiency varies from 17% to 21%. These variations of photovoltaic efficiency are mostly connected to differences in wafer technology. To make PV competitive, the price of the devices needs to be reduced further, which means ongoing research on cell structures, materials used, and technology. This research should focus on reducing the production costs, on increasing cell efficiency, on using cheaper growth technologies, and on eliminating harmful chemicals.

A promising candidate for inexpensive and environmentally friendly solar cells are cells based on zinc oxide (ZnO). ZnO thin films can be obtained using many technologies, including molecular beam epitaxy, RF magnetron sputtering, pulsed laser deposition, chemical vapor deposition, and atomic layer deposition (ALD) [3]. ALD attracts the attention of many research groups. This technology was invented in the 1970s by Tuomo Suntola from Finland [4]. Thanks to the unique properties of this technology, materials produced by ALD quickly found a number of applications in PV. For example, ultrathin films of aluminium oxide (Al_2_O_3_) are used to passivate silicon during manufacture [5]. Moreover, thin films of zinc oxide, aluminium-doped zinc oxide (AZO), and/or gallium-doped ZnO (GZO) have been successfully used in organic [6], perovskite [7], and CIGS [8] solar cells. Thanks to excellent electrical and optical parameters of AZO and GZO, these films are inexpensive alternatives for indium tin oxide (ITO) [9].

In this work, we used ALD to deposit zinc oxide nanoseeds, magnesium-doped zinc oxide (MZO) layers and aluminium-doped zinc oxide films. We thus continue our interest on photovoltaic structures based on thin films of ZnO. Recently, there have been several articles reporting the photovoltaic effect for the n-type ZnO/p-type Si heterojunction [10–14]. In several works, open-circuit voltage (*V*_OC_), short-circuit current (*J*_SC_), fill factor (FF), and photovoltaic efficiency (Eff.) were reported for ZnO/Si solar cells. Such results were published by Aqab et al. (*J*_SC_ = 19.2 mA·cm^−2^; *V*_OC_ = 67 mV; FF = 35.7%; Eff. = 0.46%) [10], Shen et al. (*J*_SC_ = 17.3 mA·cm^−2^; *V*_OC_ = 400 mV; FF = 16.5%; Eff. = 1.14%) [11], Kozarsky et al. (*J*_SC_ = 28.2 mA·cm^−2^; *V*_OC_ = 360 mV; Eff. = 5.91%) [12], and Ismail et al. (*J*_SC_ = 25 mA·cm^−2^; *V*_OC_ = 375 mV; FF = 72%; Eff. = 6.7%) [13]. In our previous work, we reported *J*_SC_ = 32 mA·cm^−2^; *V*_OC_ = 470 mV; FF = 69%; Eff. = 10.5%, and *J*_SC_ = 38 mA·cm^−2^; *V*_OC_ = 520 mV; FF = 71%; Eff. = 14% for planar and textured Zn_1−_*_x_*Mg*_x_*O/Si solar cells, respectively [14]. A solar efficiency of up to 14% was reported by us for structures grown on a 180 μm thick p-type Si substrate. Further cost reduction requires the use of a thinner Si substrate/absorber. Thus, in this paper we report photovoltaic results for a 50 μm thick Si absorber.

## Experimental

### Silicon preparation

The p-type silicon wafer with thickness of 50 μm and a diameter of 5 cm was used as absorber of the solar spectrum. Carrier concentration, mobility, and resistivity were 4.3 × 10^15^ cm^−3^, 270 cm^2^·V^−1^·s^−1^, and 5.3 Ω·cm, respectively. The silicon wafer was cut into small square pieces, ca. 1.5 × 1.5 cm^2^ in size. Samples were cleaned in acetone, iso-propanol, and twice in deionized water. The cleaning process removed impurities from the silicon surface (e.g., fat, powders, and dust). The cleaning process was carried out in an ultrasonic cleaner for 5 min for each of the chemicals. Then, the samples were dried in a stream of nitrogen. Following this stage, aluminium was deposited as a low-resistivity ohmic contact via sputtering. To improve the contact parameters, the samples were annealed at 500 °C for 5 min in argon atmosphere via rapid thermal processing. Si/Al substrates were prepared in two different ways, A and B. On the surface of sample A, zinc oxide nanorods (ZnO_NR_) were grown via a hydrothermal method (HT) to create a rough surface morphology. Sample B was placed into the ALD growth chamber without modification of the surface morphology. During HT growth, sample B was kept under nitrogen atmosphere. After the HT process, both samples were transferred to the ALD reactor.

### Hydrothermal growth

Sample A was placed into the ALD growth chamber to deposit nanoseeds of ZnO. Nanoseeds were obtained by repeating the ALD cycle for 13 times (see below in Table 1). The temperature was set to 100 °C. In the process, diethylzinc (DEZ, CAS Number 557-20-0) and deionized water were used as zinc and oxygen precursors, respectively. After growth of the ZnO nanoseeds, the samples were transferred to the HT reactor. The solution for hydrothermal growth was prepared by dissolving 3 g of zinc acetate (CAS Number 557-34-6) in 70 mL of deionized water, adjusted to pH 7.5 by a 1 mol solution of sodium hydroxide (CAS Number 1310-73-2). The hydrothermal processes of ZnO_NR_ were carried out at 70 °C for 4 min (at a microwave power level of 500 W). Monocrystalline ZnO_NR_ were grown with controlled height, width, and density. Detailed information on the HT growth is available elsewhere [15].

### Zn_1−_*_x_*Mg*_x_*O and Zn_1−_*_x_*Al*_x_*O grown by ALD

Sample A, covered by ZnO_NR_, and sample B were placed in the growth chamber of the ALD reactor. Before the growth process, the ALD reactor chamber was heated to 250 °C. The samples stayed in the chamber at this temperature for 2 h. A thin film of MZO was deposited on top of samples A and B at the same temperature. Bis(methylcyclopentadienyl)magnesium (CAS Number 40672-08-0) was used as the magnesium precursor. The positive impact of Mg on the operation of the ZnO/Si photovoltaic cells was published elsewhere [16]. The authors of this reference revealed improved electron collection by band offset engineering. On top of the solar structure, AZO was deposited as a transparent contact [17–18]. Trimethylaluminium (TMA, CAS Number 75-24-1) was used as the Al precursor. In the ALD processes, high-purity nitrogen (purity 99.999%) was used as the carrier gas. The pressure in the chamber was kept at ca. 3 × 10^−1^ Torr, using a rotary vane pump. All ALD growth parameters are summarized in Table 1.

**Table 1:** Growth parameters of ALD films.

Film	Reagent	Pulse [s]	Purge [s]	Number of ALD cycles	

ZnO nanoseeds	DEZ	0.04	8		}13×
	H_2_O	0.02	22		

MZO	Mg	0.1	8	}1×	}25×
	H_2_O	0.02	8		
	DEZ	0.04	8	}24×	
	H_2_O	0.02	22		

AZO	TMA	0.02	8	}1×	}25×
	H_2_O	0.02	8		
	DEZ	0.04	8	}24×	
	H_2_O	0.02	22		

**Figure 1:**
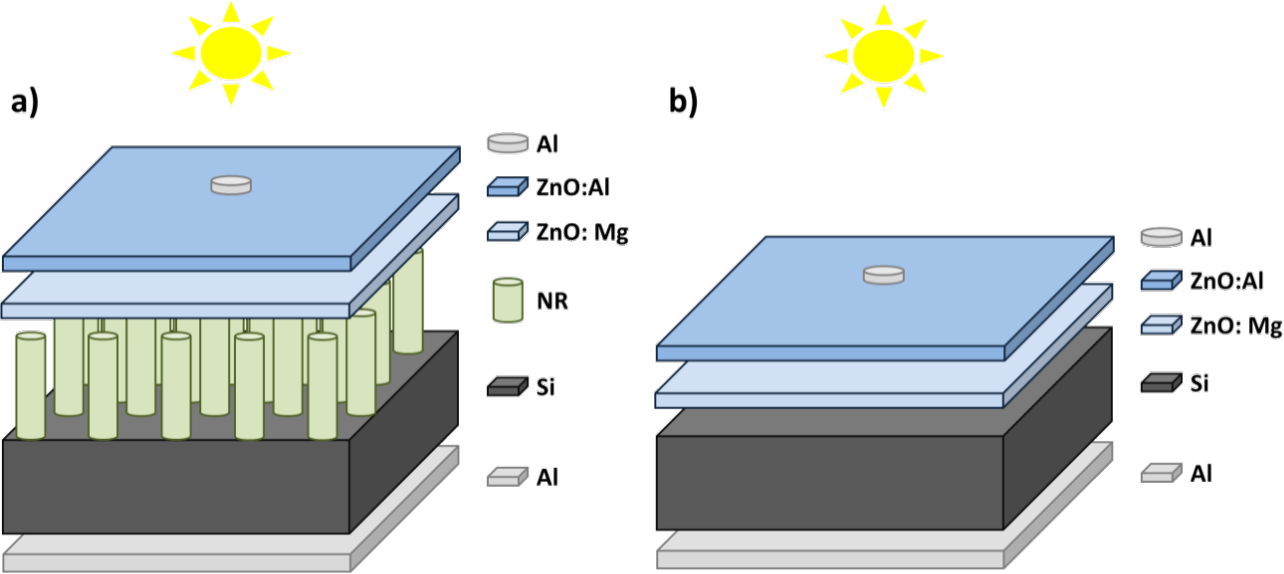
Schematic images of final PV structures studied in this work: (a) SC with ZnO_NR_, (b) planar SC.

After MZO and AZO growth, samples A and B were cut into square pieces of 0.5 × 0.5 cm^2^ in size (i.e., the total cell area equals to 0.25 cm^2^). A metal mask with hole of 0.1 cm in diameter was placed on the samples. Then, Al was deposited on top via sputtering. The simple point contact was used on top of the structure. To improve the light collection from full-size ZnO/Si SCs, grid-like contacts should be used. The resulting solar cell structures are shown in Figure 1.

## Results and Discussion

**Figure 2:**
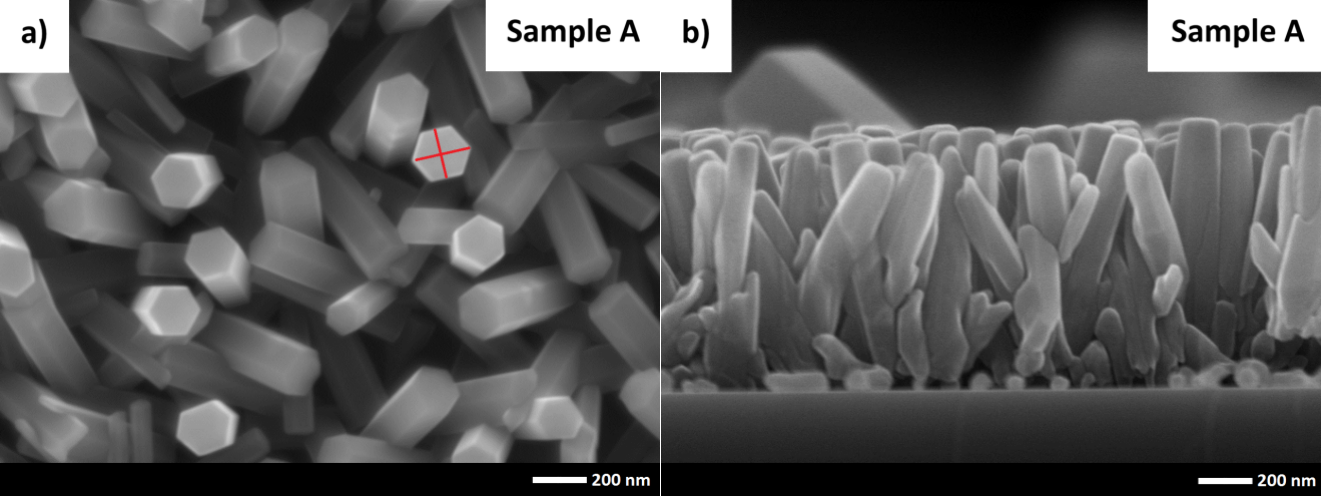
Scanning electron microscopy images of zinc oxide nanorods on the silicon surface: (a) top view, (b) cross section.

Figure 2 shows as-grown zinc oxide nanorods on the silicon surface grown by the hydrothermal method described above. Scanning electron microscopy (SEM) images revealed that the shape of the nanorods is hexagonal. The estimated width of hexagonal rods equals 160 ± 5 nm and 185 ± 5 nm, respectively. The values were measured for the red lines in Figure 2a. The height of the nanorods was 1000 ± 50 nm. There are also visible nanorods of a much smaller size, but their concentration is low. Probably the growth of the small rods started in a late stage of the process, when there was less space for their horizontal growth. The free space between the rods was very limited. Near the surface of the silicon, the nanorods are closely packed. With increasing distance from the surface the space between the nanorods increases. This effect automatically creates a rough texture, allowing us to construct a 3D top electrode and to improve light collection/harvesting in cells with such electrodes.

**Figure 3:**
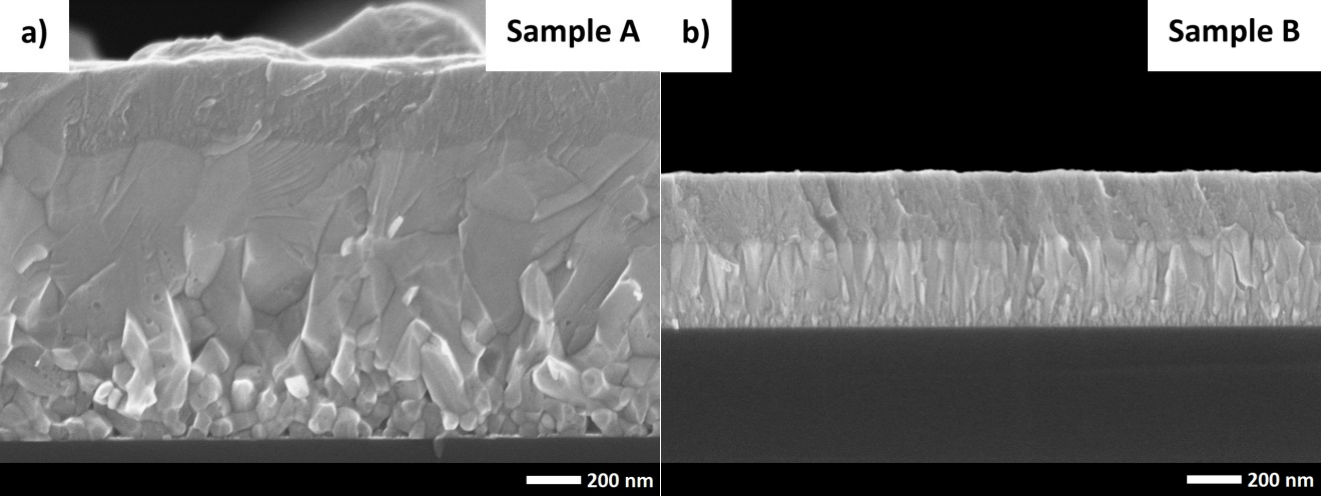
SEM images of ZnO/Si solar cells: (a) modified with nanorods, (b) planar.

Figure 3 shows cross sections of the studied photovoltaic structures. The cell labeled as sample A contains ZnO_NR_ grown via the hydrothermal method. On the nanorods, an MZO film was deposited via ALD. The use of ALD here is very advantageous, since this technique allows for a uniform and conformal coating of the rods. In the same ALD process, MZO was deposited on a polished silicon substrate (sample B). Energy-dispersive X-ray spectroscopy was used to estimate the atomic concentrations of Zn, Mg, and O in the ZnMgO films. These concentrations were (52.0 ± 0.5)%, (46.6 ± 0.5)%, and (1.4 ± 0.5)% for zinc, oxygen, and magnesium, respectively. Aluminium-doped zinc oxide films were grown on top of the samples A and B as a transparent top electrode. Comparison of the cross sections reveals the influence of the ZnO_NR_ on the growth of the magnesium-doped zinc oxide. For sample A, large mono-columns of MZO are observed. Mono-columns remained visible until their contact with the AZO film. The top surface is rough as intended, which was crucial to improve cell efficiency, especially at large angles between cell and light source. The magnesium-doped zinc oxide films exhibited grain growth mode on sample B. Close to the silicon surface, only small grains were grown initially. The size of the MZO grains varies but does not exceed a value of 50 nm. Then (for larger distances from the Si substrate), the growth mode is similar to that observed for sample A. However, the size of the columns is smaller and does not exceed 100 nm in width.

In the tested samples, a microcrystalline structure of the MZO film was detected. Our previous research proved the monocrystalline quality of ZnO_NR_ [15]. Thus, ZnO_NR_ may nucleate the growth of the ALD MZO film with a better crystalline quality, that is, the ALD films grown on nanorods have a more crystalline structure than the MZO films deposited on a clean silicon surface. In fact, our previous study showed the possibility to deposit monocrystalline ZnO films via ALD at higher growth temperatures or with a lattice-matched substrate [19].

The average thickness of the deposited films was calculated from the cross sections shown in Figure 3. It was hard to see the difference between MZO and ZnO_NR_ for sample A. Therefore, the average height was calculated for the MZO/ZnO_NR_ system, which equals to 1150 ± 50 nm. The estimated thickness of the AZO top electrode was 270 ± 20 nm. In the case of the sample B, the thickness of the deposited films equals to 320 ± 30 nm and 260 ± 10 nm for MZO and AZO, respectively.

**Figure 4:**
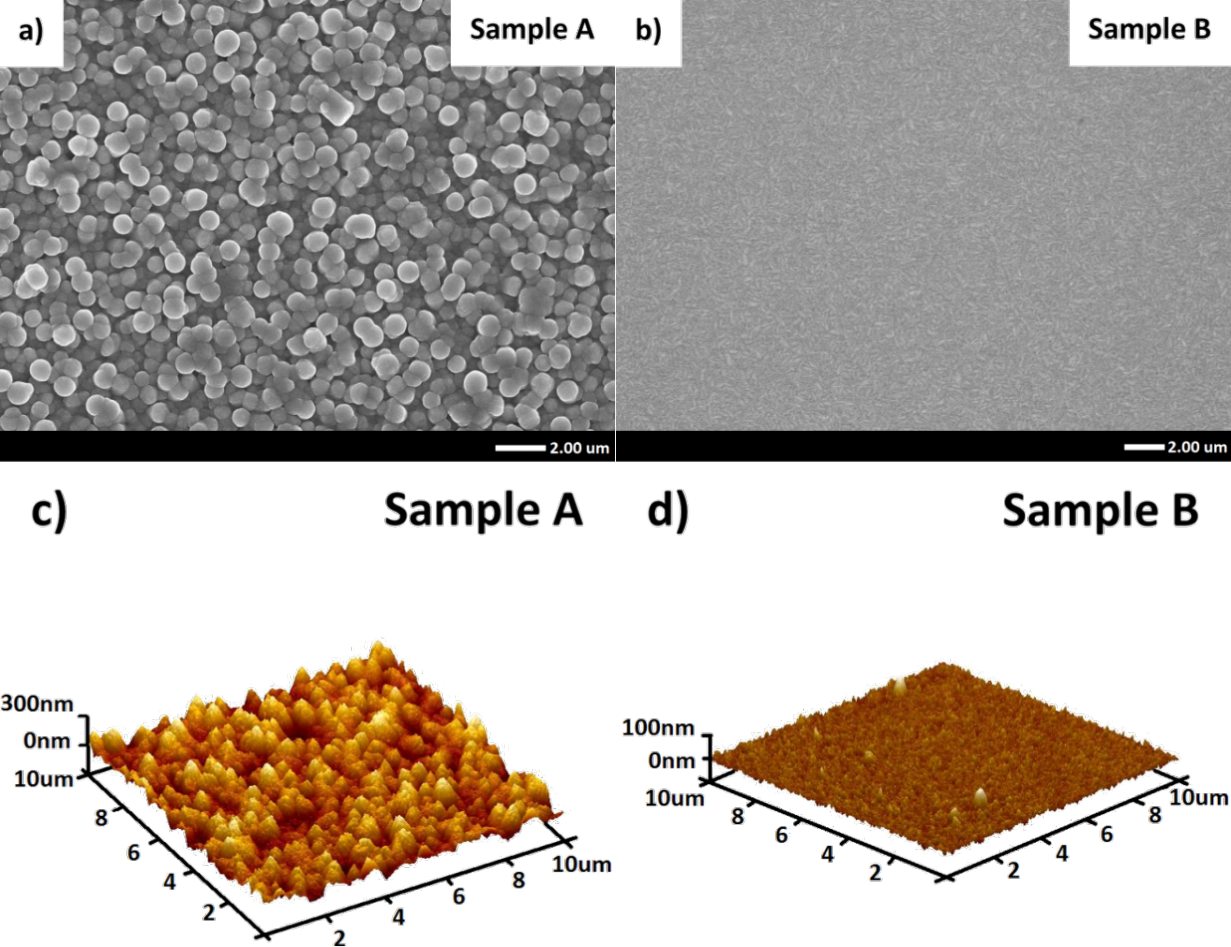
Surface morphology of (a, c) textured and (b, d) planar solar cells studied by SEM (top row) and AFM (bottom row).

Figure 4 shows the surface morphology of the tested PV cells, as measured with atomic force microscopy (AFM). The results for the photovoltaic cell modified with zinc oxide nanorods are shown in Figure 4a and Figure 4c. The results for the planar cell are shown in Figure 4b and Figure 4d. There are significant differences in the roughness, confirming the validity of our approach. A textured surface was obtained for the cell modified with ZnO_NR_. As mentioned earlier, the nanorods were separated at the top. This means that the ALD films were deposited both on the top of and in the space between the nanorods. Therefore, the deposition process led to a non-uniform surface morphology, as intended by us. The average root mean square (RMS) roughness of sample A equals 60 nm. As a result, light reflection is reduced for the cell. This effect was published previously [14] for PV structures prepared on a thicker Si absorber. This feature enhances the light-trapping effect. The impact of light-trapping on the operation of solar cells is presented by means of current–voltage curves. In contrast, sample B showed a flat surface morphology. The average RMS roughness value was 9 nm. Therefore, interference peaks in external quantum efficiency (EQE) measurements were visible.

**Figure 5:**
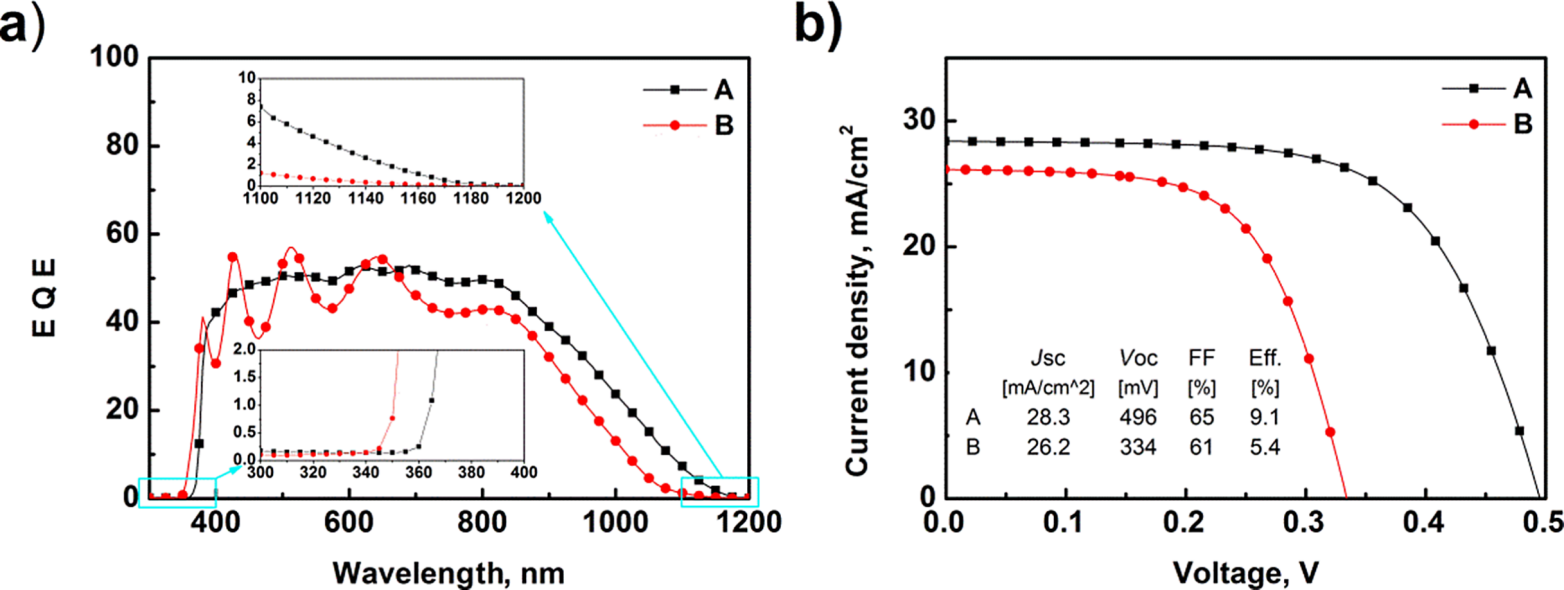
(a) External quantum efficiency and (b) current voltage characteristics of ZnO/Si solar cells.

Figure 5a shows the EQE as a function of the wavelength. The measurements were carried out in the wavelength range from 300 to 1200 nm. For photon energies greater than the ZnO bandgap, significant contributions to the external quantum efficiency were not observed. High-energy photons were absorbed close to the surface of the solar cells. Due to the high concentration of free n-type carriers, the generated electron–hole pairs (e–h) recombine very quickly (efficient Auger effect). The EQE measurement confirmed that the ZnO films work as a transparent emitter (n-type partner for silicon). For photon energies lower than the ZnO bandgap, the rapid increase of the EQE value was observed. The photovoltaic cell effectively separates e–h pairs in the wavelength range from 370 to 850 nm. The average EQE values were at the level of 50%. There were clearly visible interference peaks for sample B. In the infrared region (850–1200 nm) the largest losses in EQE were observed. Photons with a low energy have a reduced absorption probability. Hence, low-energy photons generate e–h pairs mostly deep in the junction. Such pairs (excitons) should pass through the cell before reaching the junction where they are effectively separated or recombined at the rear contact. Generally, in the infrared region absorption coefficient is low for silicon substrates [20]. Moreover, the electron–hole pairs generated deep in a cell are strongly attracted by the rear contact, and these pairs recombine without separation. This is why the external quantum efficiency drops significantly in the wavelength region of 850–1200 nm. One of the methods of improving the operation of ZnO/Si cells in the infrared range is the “passivated emitter and rear cell” (PERC) technology [21–22]. Thin films of Al_2_O_3_ or hafnium dioxide can be used as passivation film, which is deposited on the rear side of the silicon substrate. In such structures e–h pairs generated deep in a cell are separated more efficiently. The effect of rear passivation will be a topic of our further study how to improve the operation of ZnO/Si solar cells.

Figure 5b presents the current–voltage characteristics of the textured and planar ZnO/Si solar cells. Both types of the samples were investigated under standard test conditions (100 mW·cm^−2^, AM 1.5G, 25 °C). A better performance was observed for sample A. Short-circuit current values were 28.3 mA·cm^−2^ and 26.2 mA·cm^−2^ for samples A and B, respectively. The higher *J*sc value for sample A is connected to the developed surface morphology. Planar cells (sample B) reflected more incident light. Since the thickness of the absorber is small (50 μm), large current losses were observed. Comparing the results with our previous work [14], we observe a decrease in current of approximately 10 mA·cm^−2^ (a 180 μm Si wafer was used in our earlier study). The biggest differences occurred in the open-circuit voltage values. The values for sample A and sample B were 496 mV and 334 mV, respectively. The difference is related to the presence of ZnO_NR_ in the structure. Zinc oxide nanorods are monocrystalline and form a better junction with the silicon substrate (with less defects, i.e., less recombination centers). The impact of ZnO_NR_ on recombination can be observed considering the *R*_sh_ value. If *R*_sh_ increases, the cell “works” better. In the values of series resistance and fill factor, no significant differences were observed. The basic operation parameters are summarized in Table 2.

**Table 2:** Basic parameters of ZnO/Si solar cells extracted from a double exponential model.

Sample	*J*_SC_ [mA·cm^−2^]	*V*_OC_ [mV]	*R*_sh_ [Ω·cm^2^]	*R*_s_ [Ω·cm^2^]	FF [%]	Eff. [%]

A	28.3	496	1392	1.6	65	9.1
B	26.2	334	1058	1.2	61	5.4

**Figure 6:**
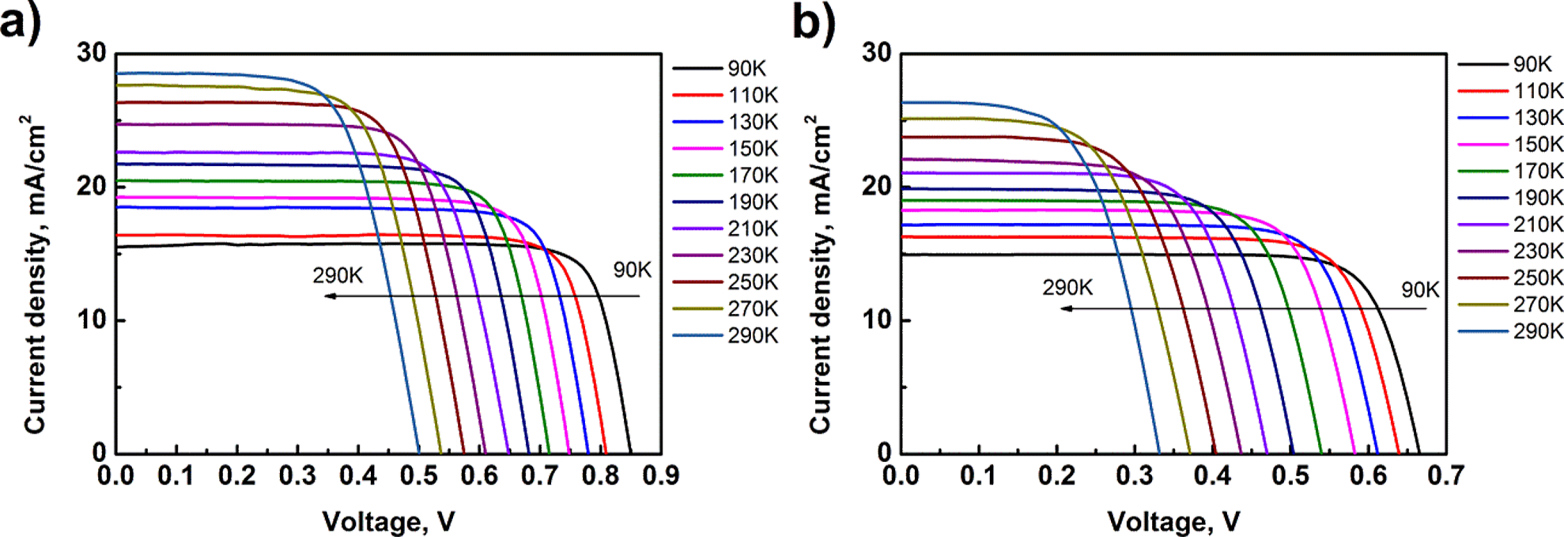
Current–voltage–temperature characteristics of (a) textured and (b) planar ZnO/Si solar cells.

Current–voltage–temperature (*J*–*V*–*T*) characteristics are presented in Figure 6. The PV structures were tested in the temperature range from 90 to 290 K with steps of 20 K. During the test, solar cells were mounted on a cold finger inside a liquid nitrogen vacuum cryostat. A transparent window in the cryostat allowed for the illumination of the cells. Solar cells with ZnO_NR_ revealed better operation parameters. A noticeable difference was observed in the open-circuit voltage.

**Figure 7:**
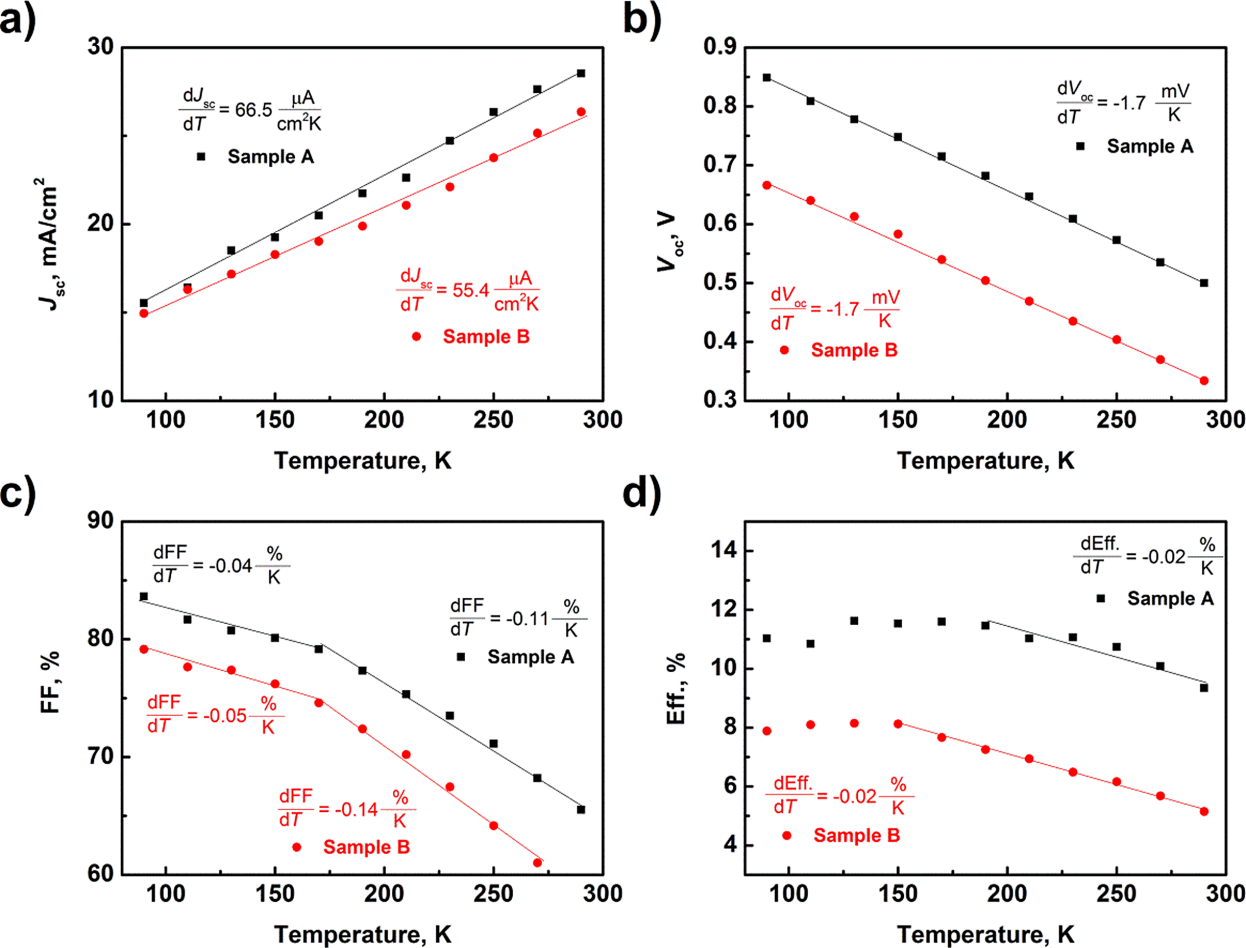
Temperature dependence of basics solar cell parameters.

The basic SC parameters *J*_SC_, *V*_OC_, FF, and Eff. as functions of the temperature are presented in Figure 7. According to [23–24], temperature has a relevant impact on semiconductor properties and the operation of solar cells. The most important equations for solar cell parameters as functions of the temperature are [24]:


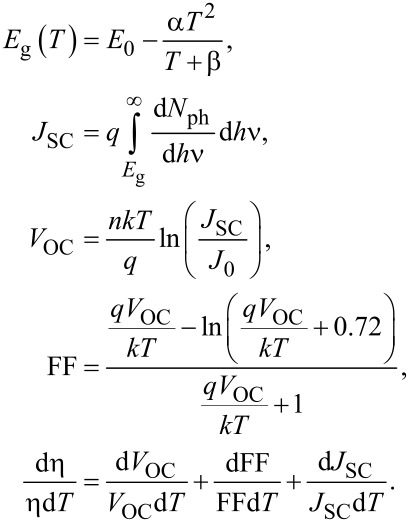


From the above equations, if temperature increases, *V*_OC_, FF and Eff. should decrease. Only an increase of *J*_SC_ should be observed.

The results in Figure 7 are in good agreement with theoretical predictions. The short-circuit current increases with temperature, while the value of the coefficient d*J*_SC_/d*T* is higher for the textured structure (sample A) and equals 66.5 μA·cm^−2^·K. For *V*_OC_ the variation of the curves is similar. Due to the existence of monocrystalline ZnO_NR_, the starting value of *V*_OC_ is higher for sample A. This effect has a direct impact on the final efficiency of the SC. Concerning the coefficient FF, there are two ranges. In the temperature range from 90 to 170 K, dFF/d*T* is ca. 0.004% per kelvin. A slightly larger value is observed for sample B. In the temperature range from 170 to 290 K this coefficient increases and is higher for sample B. The change of cells coefficients has an impact on the final temperature efficiency of the solar cells. In the low-temperature region, Eff. is stable, or only a little drop is observed. It is related to a small variation of FF in this region. In the region from 170 to 290 K, a decrease of Eff. is visible. The highest efficiencies are 11.6% (170 K) and 8.1% (150 K) for sample A and sample B, respectively. Due to the lack of similar studies in the literature, we cannot compare the obtained results with other studies of ZnO/Si solar cells.

## Conclusion

In this work, thin zinc oxide/silicon solar cells were investigated. Doped zinc oxide films (AZO, MZO) were grown via ALD and ZnO_NR_ were obtained via hydrothermal method. As substrate (absorber), a 50 μm thick silicon wafer was used. Textured and planar solar cells were constructed on the silicon surface. Solar cells with zinc oxide nanorods showed increased surface roughness. The SEM images revealed grain/columnar-like structures for both cells. In sample A, the grains were larger than in sample B. We observed a significant impact of ZnO_NR_ on the size of grains/columns. The solar cell operation was compared. Because of the 3D morphology, sample A yielded a higher *J*_SC_ value and, because of the presence of monocrystalline ZnO_NR_, *V*_OC_ was also higher. Solar cells exhibited efficiencies of 9.1% and 5.4% for the textured and planar structures, respectively. Current–voltage–temperature measurements were performed, which were in good agreement with theoretical predictions. The highest efficiencies (obtained at a reduced temperature) were 11.6% and 8.1% for sample A and sample B, respectively. Due to the lack of reports on *J*–*V*–*T* measurements in the literature, the results of these investigations cannot be compared with other studies.
